# Novel Mucoadhesive Wafers for Treating Local Vaginal Infections

**DOI:** 10.3390/biomedicines10123036

**Published:** 2022-11-24

**Authors:** Ahmed Alzainy, Joshua Boateng

**Affiliations:** School of Science, Faculty of Engineering and Science, University of Greenwich at Medway, Central Avenue, Chatham Maritime, Chatham, City of Canterbury ME4 4TB, UK

**Keywords:** Carbopol, carrageenan, metronidazole, mucoadhesion, sodium alginate, vaginal infection, wafers

## Abstract

Current vaginal formulations, such as gels and pessaries, have limitations, including poor retention. Therefore, the use of mucoadhesive formulations that adhere to the vaginal wall would allow prolonged retention and controlled drug release while reducing the required dose and the potential toxicity associated with high drug loading. The aim of the current research was to develop, characterize, and optimize freeze-dried wafers loaded with metronidazole (MTz) to treat vaginal bacterial infections. Blank (BLK) composite wafers comprising carrageenan (CARR) and sodium alginate (SA) were initially formulated; however, due to poor physico-chemical properties, Carbopol (CARB), hydroxypropylmethylcellulose (HPMC), and polyethylene glycol 200 (PEG) were included. The MTz-loaded formulations were obtained by loading optimized composite CARB:CARR- or CARB:SA-based gels (modified with HPMC and/or PEG) with 0.75% of MTz prior to freeze-drying. The physico-chemical properties were investigated using texture analysis (resistance to compressive deformation and adhesion), scanning electron microscopy (SEM), X-ray diffractometry (XRD), and attenuated total reflectance Fourier-transform infrared (ATR-FTIR) spectroscopy. Functional properties were investigated by examining the swelling, porosity, drug release, and in vitro antimicrobial activity using *E. coli* as a model infection-causative agent. The results showed that HPMC and PEG generally improved the wafer’s appearance, with smoother surfaces for easy insertion. From the physico-chemical characterization studies, only two composite wafers prepared from 8% CARB:SA 1:4 and 8% CARB:SA 1:9 gels were deemed optimal and loaded with MTz. Both formulations showed sustained drug release and achieved almost 100% cumulative release within 72 h in simulated vaginal fluid. The data obtained from the drug dissolution (release) experiments were fitted to various mathematical equations and showed the highest correlation coefficient with the Higuchi equation, suggesting a drug release based on diffusion from a swollen matrix; this was confirmed by the Korsmeyer–Peppas equation. The released MTz inhibited the growth of the *E*. *coli* used as a model bacterial organism.

## 1. Introduction

There is increasing interest in the exploitation of various mucosal routes such as the vaginal mucosa for the delivery of drugs, which are poorly absorbed via the gastrointestinal route or where local rather than systemic action is required. The vaginal environment and its physiological characteristics possess structures and specific features that help in sexual arousal and also play a role in reproduction [1]. The vagina is characterized by a dense network of blood vessels and a large surface area, which provides an ideal route for administering drugs to achieve therapeutic action, both locally and systemically [2].

To achieve systemic bioavailability, different transport (transcellular and intracellular) pathways, as well as receptor-mediated mechanisms, are involved. The key advantages of delivering drugs via the vaginal route compared to the gastrointestinal tract and skin include by-passing the first-pass metabolic effect of the liver, easy administration, and higher rates of permeation for drugs with low molecular weight [3]. However, the vaginal mucosa has not been extensively targeted due to wide individual variations that affect important parameters such as pH, as well as limited vaginal secretions that vary depending on factors such as age and the menstrual cycle. Other challenges that need to be taken into consideration when designing vaginal formulations include cultural sensitivities, hygiene, gender specificity, irritation, and the effects of intercourse. The rate and extent of absorption of drugs delivered via the vaginal route are significantly affected by changes in the thickness of the vaginal epithelium, which are caused by changes in hormone levels during the menstrual cycle. In addition, variations in endopeptidase and aminopeptidase activities cause difficulties in sustaining absorption of the administered drug [4].

The vaginal mucosa has also been shown to be a suitable site for delivering drugs that act locally within the vagina, including steroids, prostaglandins, and spermicidal, antibacterial, antiprotozoal, antifungal, and antiviral agents [5]. This notwithstanding, there are only a limited number of vaginal dosage forms currently available on the market. Vaginal formulations, such as tablets, irrigations, foams, and creams, present various disadvantages, including leaking, which causes messiness. Further, these formulations have a short residence time owing to the ability of the vaginal tract to cleanse itself, thus requiring repeated daily administration of the drug to achieve the required pharmacological action [6]. Pessaries are ovoid-shaped solid dosage forms specifically designed for vaginal administration and prepared by melting, molding, and compression. Though they are characterized by ease of production and a low cost, the inconvenience of vaginal pessaries, such as messiness upon application, poor retention in the vagina, and poor stability, are key limitations. Administered vaginal pessaries dissolve in the vaginal fluids or melt at body temperature, resulting in the rapid release of drugs; therefore, more sustained-release formulations have been proposed to circumvent this problem [7].

Various approaches for developing innovative vaginal formulations that could satisfy the needs of both patients and clinicians have been undertaken [8]. Considerable attention has been directed towards developing formulations that can control the release of a loaded drug, which can afford high amounts of a drug at therapeutic levels, and that allow for prolonged drug release, and therefore result in the need for only a single dose. Novel systems include liposomes [9], vaginal rings [10], cubic gels [11], and systems designed using polystyrene and silicone-based elastomers [8]. One group of auxiliary agents that is important for successful vaginal delivery is the mucoadhesive polymer group, which now underpins the design and development of novel systems. In recent years, attention has been directed towards various polymers that are used in the form of hydrogels to impart bioadhesive properties so that the formulations remain in the vaginal cavity long enough to elicit the intended therapeutic action. Subsequently, bioadhesive vaginal formulations have been designed that allow drug release at a controlled rate for treating both local and systemic diseases [12].

Common bioadhesive materials include natural (e.g., chitosan, carrageenan, sodium alginate, and cellulose derivatives) as well as synthetic (e.g., Carbopol) polymers. The vaginal mucus forms a cross-linked interaction with bioadhesive polymers, which causes the formulation to swell and to release the active substance in a controlled manner to achieve the desired bioavailability [13]. In addition, some bioadhesive polymers are more effective than others depending on their properties, such as their interactions with cervical mucus or the vaginal pH. Therefore, in the formulation of vaginal drug-delivery systems, the characteristics of the polymer, the vaginal environment, and the dosage form characteristics must be taken into consideration [14]. Various marketed products have used bioadhesive delivery systems for treatments, using polymers with ideal properties such as a low cost of production, avoidance of the use of solvents, ease of self-administration without using an applicator, the maintenance of structural integrity when hydrated, the absence of local irritation, quick mucoadhesion, the ability to remain in the vaginal cavity for long periods even with no physiological secretions, a reduced dosing frequency, and improved chemical and physical stability [13]. Among the natural biopolymers, chitosan is one of the most used in this regard due to its functional properties such as biocompatibility and ease of biodegradation, as well as a strong bioadhesive behavior. The latter property is attributed to its cationic nature, which allows strong ionic bond formation with mucosal surfaces [15], and this can be further enhanced via derivatization using side chains such as thiol groups. For vaginal drug delivery, chitosan’s known biological (antimicrobial and anti-inflammatory) actions make it a suitable candidate to use as a drug-delivery platform for treating local vaginal infections [16]. Fitaihi et al [17] reported on the formulation of chitosan-based vaginal tablets loaded with fluconazole. For synthetic polymers, nonoxynol-9, which is a spermicidal agent, has been formulated as a gel using Carbopol 934P as the bioadhesive polymer and succeeded in releasing high amounts of the drug within 2 min after insertion and then maintaining a controlled release over 6 h [18].

Microorganisms such as bacteria occur naturally in various parts of the body, including mucosal cell linings such as the mouth and vagina. Commonly occurring bacterial strains in the vaginal cavity include *Gardnerella*, *Lactobacilli*, *Bacteroides*, *Mobiluncus*, *Mycoplasma*, and *Streptococci.* The uncontrolled growth of these bacterial strains results in itchiness, smelly discharge, and painful intercourse, and this form of vaginal infection is known as bacterial vaginosis [19]. Vaginal infections are commonly treated using antifungal and antibacterial agents formulated in the form of tablets, gels, foams, powders, and creams. However, most of these formulations have limited residence time in the vaginal tract and therefore require an increased drug dose and increased application frequency. Based on these challenges, vaginal delivery systems must be developed and optimized to improve the retention of the dosage form and loaded drug in the genital tract over a longer period.

This study therefore involved the development and optimization of freeze-dried polymer-based wafers as platforms for delivering metronidazole via the vaginal mucosa with the aim of prolonging the residence time to achieve local therapeutic effects for the treatment of bacterial vaginosis.

## 2. Materials and Methods

### 2.1. Materials

Carbopol 974P NF and carrageenan (Gelcarin GP 379) NF were donated by IMCD Ltd. (Sutton, UK); polyethylene glycol 200, sodium alginate W201502, mucin from porcine stomach, urea, gelatin, and calcium hydroxide were purchased from Sigma-Aldrich (Gillingham, UK). Sodium chloride, potassium hydroxide, lactic acid, glucose, hydroxypropylmethyl cellulose, metronidazole (99%), bovine serum albumin, ethanol, acetic acid, acetonitrile, and glycerol were obtained from Fisher Scientific (Loughborough, UK).

### 2.2. Formulation Development of Wafers

#### 2.2.1. Blank (BLK) Wafers

Composite formulations were prepared using Carbopol (CARB) as the base polymer in all cases. CARB was mixed with sodium alginate (SA) or carrageenan (CARR) at different ratios and concentrations, while polyethylene glycol 200 (PEG) and hydroxypropylmethyl cellulose (HPMC) were also added to some of the composite formulations as modifiers to improve their physical properties as part of the formulation development process (Table 1, Table 2 and Table 3). Gels were prepared by first dissolving CARB in deionized water, and the other polymers were then added slowly to avoid lump formation. Once homogeneous gels were obtained, 3 g of each formulation was poured into Eppendorf tubes, which were placed on a rack and loaded into a freeze-drying machine. The wafers were prepared by freeze-drying the gels using a Virtis Advantage XL 70 freeze-dryer (Biopharma Process System, Winchester, UK). The freeze-dryer was programmed to undergo freezing, primary drying, and secondary drying processes in automatic mode.

#### 2.2.2. Drug-Loaded (DL) Wafers

The formulations for drug loading were selected based on the results of a physico-chemical characterization (especially hardness). The selected optimized formulations for drug loading were: (i) 6% CARB:CARR 1:3 + 1% HPMC (W14), (ii) 6% CARB:CARR 1:1 + 1% HPMC (W16), (iii) 8% CARB:SA 1:2 + 8% PEG (W20), (iv) 8% CARB:SA 1:4 (W10), and (v) 8% CARB:SA 1:9 (W13). The gels were prepared by first dissolving metronidazole (MTz) in deionized water, and then the polymers were added slowly to avoid lump formation. The composition of the MTz-loaded wafers has been summarized in Table 4. Wafers were obtained by freeze-drying the gels in Eppendorf tubes using the automated lyophilization cycle described for the BLK wafers above.

### 2.3. Physico-Chemical Characterization

#### 2.3.1. Mechanical Properties

The resistance to compressive deformation (hardness) was evaluated under ambient conditions using a TA HD Texture Analyser (Stable Microsystem Ltd., Surrey, UK) with a load cell of 5 kg. The results were processed using the Texture Exponent 32 software program (Stable Microsystem Ltd., Surrey, UK). The hardness was measured by compressing the formulations (*n* = 3) in the middle, at the tip, and vertically with the help of a 6 mm-diameter (P/6) probe using a trigger force of 0.001 N, a penetration depth of 2 mm, a test speed of 1 mm/s, and a return distance of 10 mm.

#### 2.3.2. Adhesive Properties

The mucoadhesive properties (peak adhesive force (PAF); total work of adhesion (TWA) and cohesiveness) of the lyophilized wafers were investigated using the same Texture Analyser mentioned above, but in tension mode. The wafers (*n* = 3) were attached to an adhesion rig (75 mm in diameter) using double-sided adhesive tape. The mucoadhesive surface was prepared by pouring 20 g of hot gelatin gel (6.67% *w/w*) into a Petri dish (90 mm diameter) and allowed to set by cooling. The surface of the set gelatin was then equilibrated with simulated vaginal fluid (SVF) (0.5 mL) to represent the vaginal mucosa. The probe was lowered until the attached wafer contacted the gelatin surface and was kept in place for 60 s using a force of 1 N to ensure proper contact. The probe was detached using pre-test and test speeds of 0.5 mm/s and post-test speed of 1 mm/s, a 0.05 N trigger force, and a 10 mm return distance. The force required to break the adhesive bond between the wafer and gelatin surface was determined by the maximum force (PAF). The TWA was obtained by calculating the area under the force–distance curve while the cohesiveness was the total distance (in mm) travelled by the probe before the wafer was completely detached from the gelatin surface. Each formulation was tested three times (*n* = 3).

#### 2.3.3. Dislodge Test

The dislodge test was used to estimate how long the wafers would stay in the vagina before detachment from the vaginal wall. An amount of 10 mL of SVF solution was maintained at 37 °C. The wafer was gently removed from the Eppendorf tube using a pair of tweezers. A syringe was used to transfer mucin solution (1 mL) into the Eppendorf tube, followed by 3–4 droplets of SVF solution. The Eppendorf tube was closed and shaken to allow the SVF and mucin solution to coat and wet the inside of the tube, after which the wafer was placed back into the moistened Eppendorf tube. The Eppendorf tube was inverted vertically with its cap opened and placed on a flat surface, and the narrow tip was cut off to allow for fluid to be added. At specific time intervals (1 h), 2 to 3 drops of SVF solution were introduced into the Eppendorf tube through the cut tip and regularly observed to determine when the hydrated wafer dislodged from the tube. Each formulation was tested in triplicate (*n* = 3).

#### 2.3.4. Swelling (Hydration) Capacity

The swelling capacity was measured to investigate the hydration and swelling ability of each formulation. SVF was heated to 37 °C to mimic the temperature of vaginal fluid and approximately 20 mL was transferred into a pre-weighed Petri dish. The wafers were weighed (dry weight) and then placed into the Petri dish containing SVF. The wafers and Petri dishes were then reweighed after removing the free SVF and swabbing the Petri dishes with paper towel to remove any excess SVF at 0.5, 1, 2, 3, 6, 24, 48, and 72 h intervals until total disintegration. After each weighing of the Petri dishes containing the swollen wafer, a fresh solution of SVF was added. The percentage water uptake (swelling capacity) (*n* = 3) was calculated using Equation (1) [20]:

Swelling capacity (%) = 100 × (Ws − W)/W(1)

where Ws is the weight of the hydrated wafer and W is the initial weight of the wafer.

#### 2.3.5. Scanning Electron Microscopy (SEM)

A Hitachi UHR FE-SEM SU8030 microscope (Hitachi High-Technologies, Krefeld, Germany) was used to evaluate the surface morphology of the formulations. The wafers were cut into small slices and placed onto aluminum pin-type stubs (G301) (Agar Scientific Limited, Stansted, UK) using double-sided adhesive carbon tape (G3347N) (Agar Scientific Limited, Stansted, UK). Each sample was coated with gold using an Edwards 188 Sputter Coater S1508 (Leica Microsystems (UK) Ltd., Milton Keynes, UK) and analyzed at an accelerating voltage of 5.0 kV [21]. The emission was set to 10 µA with normal probe current, and 5 condensers were used with the working distance (Z height) set to 8 mm.

#### 2.3.6. Porosity

The solvent displacement method was employed to determine the porosity of the wafer micro-structure. The wafers were accurately weighed and the weighed sample was subsequently immersed in absolute ethanol (20 mL) in a 50 mL Falcon tube. The set up was left for 3 h to ensure complete saturation and then degassed to remove all air bubbles from the wafer formulations. The wet samples were removed from the solvent, wiped gently with tissue paper to remove excess solvent, and immediately re-weighed to avoid the loss of ethanol via evaporation. The porosity (%) of each wafer formulation (*n* = 3) was calculated using Equation (2) [22]:

Porosity (%) = (W_f_ − W_i_ /ρ_e_ × V_g_) × 100 (2)

where:

V_g_ = wafer’s geometrical volume;

W_f_ = final weight of wafer;

W_i_ = initial weight of wafer;

ρe = ethanol density (0.789 g/cm^3^).

#### 2.3.7. X-ray Diffraction (XRD)

The physical form (crystalline/amorphous) of the pure starting materials and wafers was analyzed on a D8 Advance X-ray Diffractometer (Billerica, MA, USA). Before analyzing, the wafers were compressed using two clean glasses, placed in the holder, and mounted on the sample cell. For starting materials, the powders were held together using mylar before placement on the sample cell. The samples were analyzed in transmission mode with a diffraction angle range of 5° to 50° 2θ, a 0.04° step size, and a scan speed of 0.4 s/step [21].

#### 2.3.8. Attenuated Total Reflectance Fourier-Transform Infrared (ATR-FTIR) Spectroscopy

Samples were analyzed for their FTIR spectral profiles using a Perkin Elmer Spectrum instrument (Waltham, MA, USA) equipped with a diamond universal ATR unit. The wafers were cut into small pieces whilst the starting materials were used in powder form, and the samples were placed on the ATR diamond crystal. A pressure clamp was used to apply force on each sample to ensure adequate contact between the diamond crystal and the samples. The FTIR spectra were recorded for each sample (wafer and pure starting material) using a resolution of 4 cm^−1^ within the range of 400–4000 cm^−1^. Background spectra were subtracted to obtain a consistent absorbance for each sample.

### 2.4. In Vitro Drug Dissolution and Release Profiles

The in vitro drug dissolution of the MTz-loaded wafers was performed using an Agilent BIO-DIS reciprocating cylinder cell apparatus (Agilent Technologies, Cheshire, UK). The formulation was covered with a dialysis membrane with MWCO 3.5 KDa and a diameter of 29 mm and placed in chambers containing wire meshes with a pore size of 0.4 mm. The cylindrical compartment was filled with 200 mL of SVF at a pH of 4.2 ± 0.1 with the mesh on the receptor surface. The system was placed in a water bath at 37 °C and the automated chambers were dipped into the SVF at a rate of 5 dips/min. Aliquots (2 mL) of the SVF were withdrawn at predetermined time intervals using a 3 mL syringe (and replaced with the same volume of fluid at 37 °C), filtered through a 0.45 μm cellulose acetate membrane, transferred into glass vials, and analyzed by high-performance liquid chromatography (HPLC). The percentage of cumulative drug released from the wafers (*n* = 3) at each time point was calculated and plotted against time.

#### 2.4.1. HPLC Analysis

The saturation solubility of the drug within the DL wafers was investigated to determine the minimum volume of SVF needed for drug dissolution, to ensure sink conditions. This was carried out by dissolving a whole DL wafer in 50 mL of SVF for 48 h and then injecting onto the HPLC column. By using the standard calibration equation, the minimum volume of SVF needed for the drug dissolution test was calculated. The MTz released during the drug dissolution was analyzed using an Agilent 1200 HPLC system equipped with an auto sampler and data analyses were performed using a Chemstation^®^ software program (Agilent Technologies, Cheshire, UK). The stationary phase used was a HICROM S10-0DS1-3634 column (250 × 4.6 mm) with a 10 μm particle size (Waters, Leicestershire, UK). The mobile phase consisted of a mixture of acetonitrile and 0.01% trifluoroacetic acid (TFA) in the ratio 80:20 *v*/*v*. The flow rate of the mobile phase was maintained at 1.5 mL/min and the wavelength for MTz detection was set at 315 nm. A volume of 5 μL was injected during each run with a column temperature of 60 °C.

Eight calibration solutions (0.08 to 1.0 mg/mL) were prepared, analyzed in triplicate, and used to plot the linear calibration curve. The intraday accuracy and precision of the assay were evaluated by analyzing three replicates of the standard MTz calibration solutions at each concentration.

#### 2.4.2. Evaluation of Drug-Release Mechanisms

Release kinetic equations are mathematical models employed to investigate the mechanisms of drug release from different formulations. The various equations are compared to determine the model that fits the release data best, based on the model that gives the highest correlation coefficient (R^2^) [23]. In this study, four kinetic models were used to investigate the mechanism(s) of the MTz release from the wafers, as shown in Table 5.

### 2.5. Antibacterial Study

#### 2.5.1. Minimum Inhibitory and Bactericidal Concentrations

In vitro antibacterial testing of the MTz-loaded wafers was performed using turbidimetric and Kirby Bauer disk diffusion assays. Bacterial cultures were obtained in a brain heart infusion (BHI) broth medium at 37 °C using *E. coli* as the model bacterial organism. The minimum inhibitory concentrations (MICs) and minimum bactericidal concentrations (MBCs) for MTz were measured in Muller Hinton (MH) broth using the broth dilution method. Serial dilutions of MTz (4–10 mg/mL) were prepared in sterile MH broth and inoculated to achieve a final bacteria concentration of 10^6^ CFU/mL (*n* = 3). After 24 h, samples from each treatment and relevant dilutions were plated on MH agar plates, and the colony-forming units (CFUs) were counted after incubation for another 24 h at 37 °C and used to determine the MBC. The number of viable cells was plotted against the antibiotic concentration and the number of cells per ml was calculated using Equation (3):

# of cells (CFU/mL) = [(colonies counted × dilution factor)/volume plated)](3)

#### 2.5.2. Turbidimetric Assay

For the turbidimetric assay, 10 mL of a prepared *E. coli* suspension (10^6^ CFU/mL) was transferred into sterile test tubes that contained MTz-loaded wafers. Tubes that were filled with only the *E. coli* suspension and a pure MTz solution were used as the negative and positive controls, respectively. The tubes were incubated (37 °C, 180 rpm), aliquots were sampled after 1.5, 3, 6, 12, and 24 h, and the absorbance was measured (625 nm). Further, 0.1 mL aliquots were plated directly or after serial dilution to count the number of viable bacterial colonies, with only the plates having 30 to 300 colonies accepted for determining the number of CFU/mL [24]. The experiments were conducted in triplicate (*n* = 3).

#### 2.5.3. Kirby Bauer Disk Diffusion Assay

MH agar plates were prepared as previously reported [25]. Briefly, MH agar was autoclaved for 45 min at 121 °C and allowed to cool to ambient temperature, and then 25 mL was poured into a Petri dish to obtain a 5 mm layer of solid agar slant. A sterile swab was placed into the tube containing the *E. coli* suspension (10^6^ CFU/mL) and streaked over the surface of the entire MH agar plate three times with clockwise rotation. The wafers were placed at the center of the colonized agar plates and the plates were incubated at 37 °C for 24 h, after which the zone of inhibition (ZOI) in mm was measured with a Vernier caliper. A 6 mm filter disk loaded with 20 μg of pure MTz and a BLK wafer were used as the positive and negative controls, respectively.

### 2.6. Statistical Analysis

Statistical analyses were performed to compare the results of the quantitative data, including hardness, mucoadhesion, hydration capacity, and porosity, using one-way ANOVA. The results are expressed as the mean ± standard deviation and the level of significance was set at *p* < 0.05.

## 3. Results and Discussion

### 3.1. Formulation Development and Optimization of BLK and DL Lyophilized Wafers

The W1–W4 composite gels were the easiest to pour into the Eppendorf tubes and the resulting ‘rocket-shaped’ wafers were also the easiest to remove compared to W14, W16, W7, W10, W17, W19, W20, and W21, which had higher total polymer contents. The presence of HPMC in the formulations imparted a smooth outer surface to the wafers, while PEG (which acts as plasticizer) decreased the friability and improved the flexibility by increasing the spacing between polymer chains. Some composite mixtures were difficult to dissolve during formulation and were unable to form homogenous gels (data not shown). Therefore, only W1–W7, W10, W14, W16, W17, W19, W20, and W22 were evaluated for mechanical strength to confirm their physical characteristics. The formulations for MTz loading were selected based on the results of the physico-chemical characterization, especially the hardness tests, with W10, W13, W14, W16, and W17 selected as the optimized formulations for drug loading.

### 3.2. Physico-Chemical Characterization

#### 3.2.1. Mechanical Properties of Wafers

**Figure 1:**
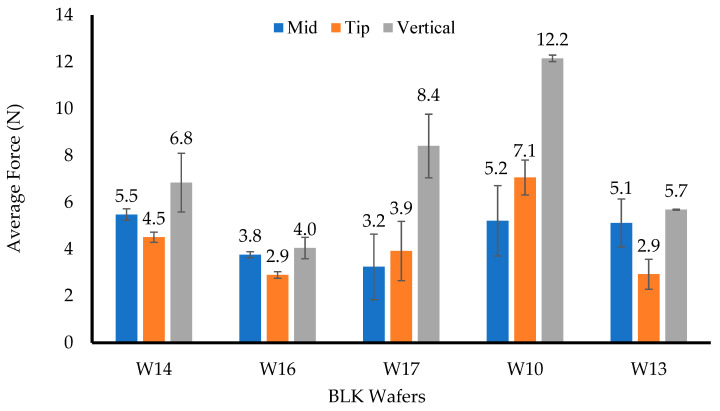

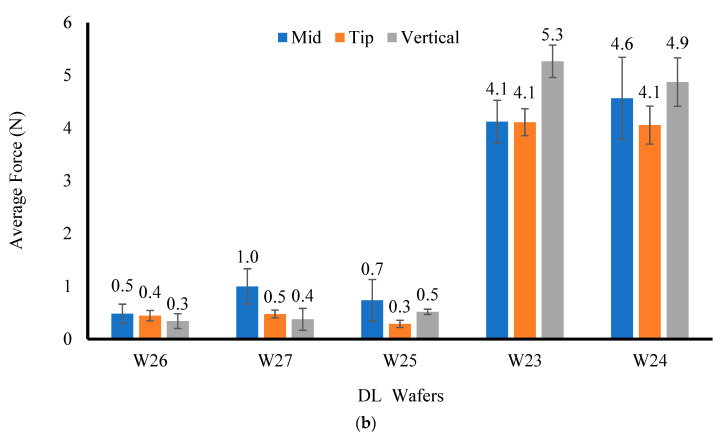
Mechanical properties of BLK (**a**) and DL (**b**) wafers.

Figure 1 shows the vertical hardness profiles for the (a) BLK and (b) DL wafers. The W14 and W16 wafers showed hardness values of 6.8 ± 1.3 N and 4 ± 0.13 N, respectively, with slight decrease in the hardness of the W16 wafer, which was less than 5 N, but it was expected that the hardness will increase when loaded with a drug. The W10 and W13 wafers showed vertical hardness values of 12.2 ± 0.14 N and 5.7 ± 0.02 N, respectively, while the W17 wafer showed a vertical hardness value of 8.4 ± 1.4 N.

The ideal value reported for hardness is between 3 and 6 N [26], although this depends on other factors such as the type and number of polymers, as well as other excipients used to prepare the final formulations. However, vaginal wafers need much more than the reported values; therefore, it was hypothesized that hardness values between 5 and 8 N would be better, considering the need for the wafers to be able to resist pressing or pushing deformation forces during insertion. The MTz-loaded wafers (W25, W26, and W27) showed average values below 5 N, while W10 and W13 showed hardness values of 5.27 ± 0.31 N and 4.82 ± 0.46 N, respectively. Therefore, most of the MTz-loaded wafers were deemed too weak with the exception of those obtained from 8% gels (W10 and W13), which showed good hardness profiles overall.

Compared to the results of the corresponding BLK wafers, the hardness values decreased after loading MTz for all formulations. This decrease was attributed to the decreased porosity of the wafers due to the added drug and recrystallization on the pore walls. Statistical analysis of the hardness results for the DL wafers showed that the hardness values of W25, W26, and W27 were significantly different (*p* < 0.05). This difference might be attributed to the total polymer concentration of the samples, which resulted in increased viscosity due to the increased density of the starting gels. For W25, the weakness could be attributed to a higher concentration of CARB, which meant that SA had to be reduced, as well as the presence of PEG, which acts as plasticizer to reduce the brittleness of the formulation. The W23 and W24 wafers showed a *p*-value of 0.06, which implies no significant difference between these two formulations.

#### 3.2.2. Adhesive Properties

**Figure 2:**
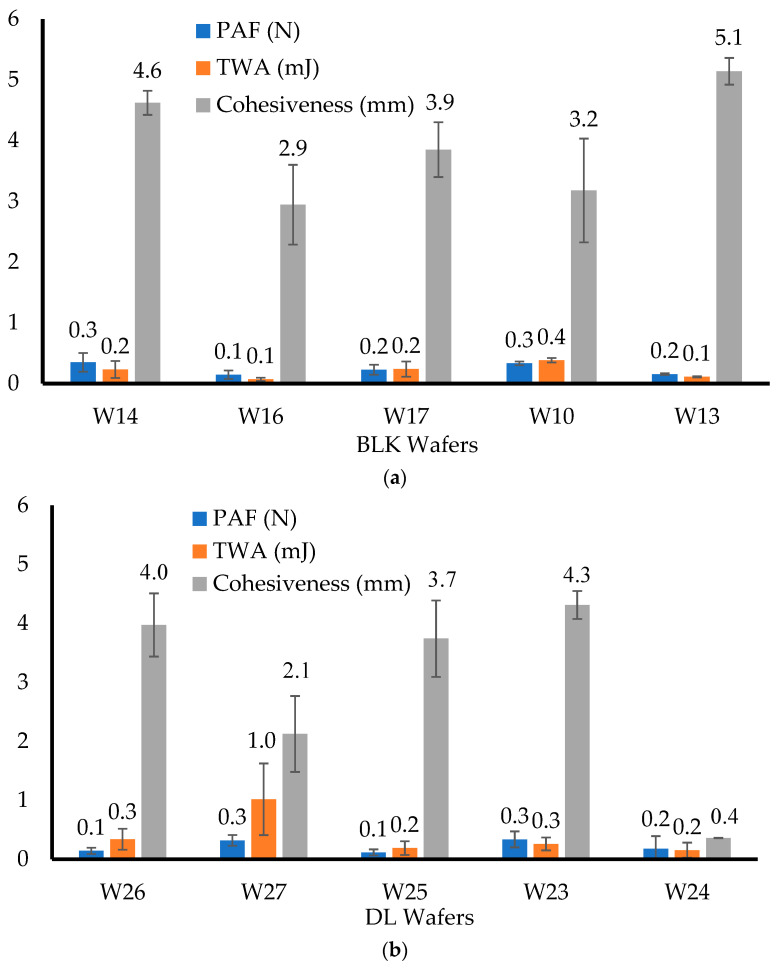
In vitro adhesive properties of BLK (**a**) and DL (**b**) wafers. Data labels on the histograms have been rounded to 1 decimal place.

Figure 2a showed that the W14 wafer had a PAF of 0.35 ± 0.16 N, while W16 showed the lowest PAF value of 0.14 ± 0.07 N. The TWA value of the W14 wafer was 0.23 ± 0.14 mJ, while W16 showed the lowest value of TWA at 0.07 ± 0.03 mJ. The W14 wafer showed a cohesiveness of 4.62 ± 0.2 mm, while W16 showed the lowest value of cohesiveness at 2.94 ± 0.66 mm.

The W10 and W13 wafers showed a PAF of 0.33 ± 0.03 N and 0.18 ± 0.03 N, respectively. The TWA of the W10 and W13 wafers showed values of 0.38 ± 0.04 mJ and 0.1 ± 0.01 mJ, respectively, while the cohesiveness of the W10 and W13 wafers were 3.18 ± 0.86 mm and 5.1 ± 0.22 mm, respectively. W17 had a PAF value of 0.23 ± 0.08 N, a TWA of 0.24 ± 0.13 mJ, and a cohesiveness value of 3.85 ± 0.45 mm. This showed that with an increasing concentration of CARB, the cohesiveness decreased. The TWA values of the CARB:CARR wafers were higher than those of the CARB:SA wafers due to higher amounts of CARR in the formulations. This imparts adhesive properties due to the anionic structure of CARR, which helped the wafer to adhere more efficiently to the model mucosal surface. In addition, anionic polymers that possess sulfate groups can bind to mucosal tissues better than those with carboxylic acid [27]. Therefore, CARR increased the mucoadhesive performance due to the negative charge of its sulfate group, which formed ionic bonds with the positively charged mucin present on the model mucosal surface [28]. The cohesiveness was also affected by the different CARB, CARR, and SA ratios, as well as by the total polymer content. The statistical analysis showed that there was a significant difference between all the wafers (*p* < 0.05) for the PAF, TWA, and cohesiveness, as they showed *p*-values of 0.002, 0.01, and 0.04, respectively.

All the wafers showed an extremely low PAF and a low hydration ability, but a high cohesiveness resulting from the formation of a gel-like structure upon hydration. According to Tobyn et al [29], a high ionic strength coupled with sodium and potassium ions present in the media leads to a decrease in adhesion when the amount of CARR is higher in the formulation. Figure 2b shows that the DL wafers had a low PAF, with the W23 and W27 formulations showing higher values of PAF at 0.32 ± 0.09 N and 0.34 ± 0.14 N, respectively, while the W26, W25, and W24 wafers showed the lowest PAF values at 0.14 ± 0.05 N, 0.11 ± 0.05 N, and 0.18 ± 0.21 N, respectively. The TWA values of the W26, W25, W23, and W24 wafers were 0.34 ± 0.18 mJ, 0.11 ± 0.12 mJ, 0.26 ± 0.11 mJ, and 0.15 ± 0.13 mJ, respectively, while the W27 wafer showed a higher TWA at 1.02 ± 0.61 mJ. The cohesiveness values for the DL wafers were also affected by the different ratios and total polymer content. The W24 wafer showed the lowest cohesiveness at 0.36 ± 0.01 mm due to its high concentration of SA, while W26, W27, W25, and W23 showed higher cohesiveness values of 3.97 ± 0.54 mm, 2.13 ± 0.64 mm, 3.74 ± 0.65 mm, and 4.31 ± 0.24 mm, respectively.

Compared to the results of the corresponding BLK wafers, the PAF, TWA, and cohesiveness decreased after loading the MTz for all formulations except W23, which showed an increase in cohesiveness. The reason for this may be because the SA wafers were very soft, and when 1 N of force was applied, the wafers were squashed, thus increasing the distance travelled before detachment. The statistical analysis showed that there was a significant difference between the DL wafers for the PAF, cohesiveness, and TWA (*p* < 0.05).

#### 3.2.3. Dislodge Test

The adhesion test performed on a horizontal gelatin gel, though indicative, was not completely representative of what would be encountered upon insertion of a wafer into the vaginal cavity. The dislodge test examined the ability of the wafer to attach itself onto a cylindrical surface and how long it remained in a vertical position before detaching from the surface. All the wafers remained adhered to the wall of the moistened Eppendorf tube throughout the 6 h experimental test period. Once the wafers made contact with the SVF on the inside wall of the Eppendorf tube, the wafers hydrated and formed adhesive interactions with the inner wall of the Eppendorf tube, which prevented them from being dislodged.

**Figure 3:**
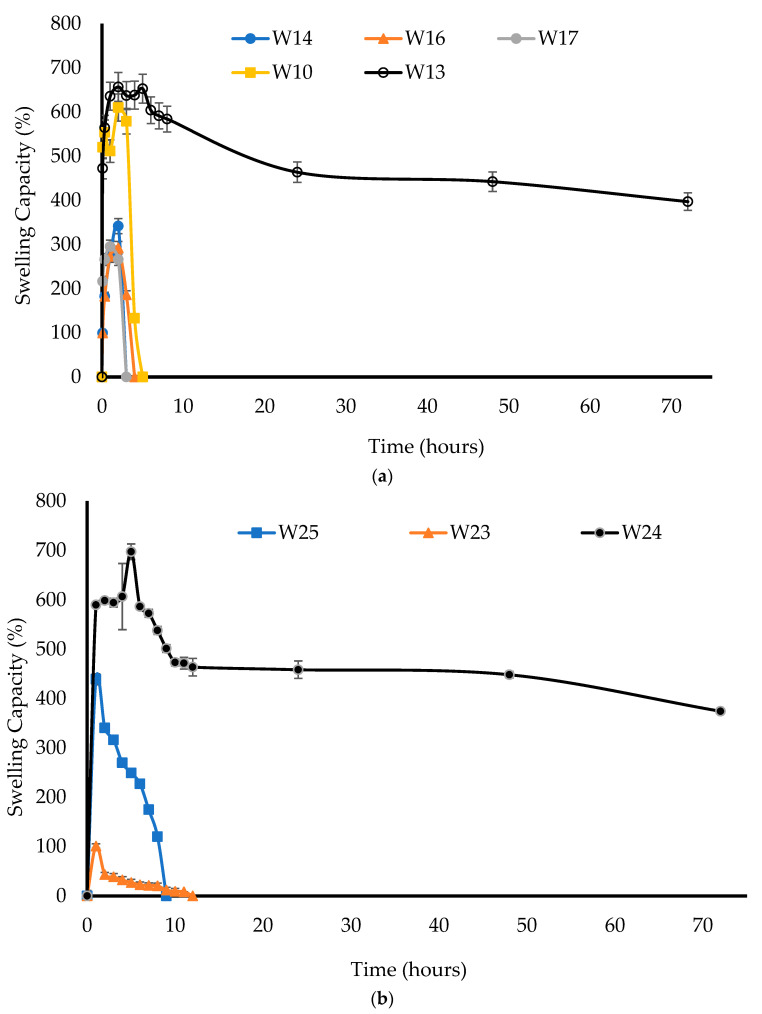
Swelling index of BLK (**a**) and DL (**b**) wafers.

The W26 wafers completely shrunk after 3 and 5 h, respectively, and this confirmed the low PAF and TWA in Figure 2 and the low swelling capacity in Figure 3 below. This showed that the loading of MTz lowered the adhesion compared to the BLK wafers. This is because the increased amount of CARB in the wafers formed a gel-like structure upon hydration and enhanced the adhesive interactions with the substrate, aided by the presence of sodium and potassium ions [29]. The presence of MTz on the pore walls of the wafers (see SEM section) reduced the contact of the polymers present within the wafers, which could be the main reason for the reduction in adhesion for the DL wafers compared to the BLK wafers.

#### 3.2.4. Hydration Capacity (Swelling Test)

Figure 3a showed that increasing the total polymer concentration of the initial composite gels to 6% resulted in an increase in the swelling capacity, and the resulting wafers only disintegrated after 5 h, as they had high hardness values and sub-pores.

This shows that these formulations have potential for a more prolonged drug delivery. For example, the W10 wafer had a swelling capacity of 610 ± 64% and completely disintegrated after 5 h, while the W13 wafer had a swelling capacity of 657 ± 2.9% and lasted until 72 h, implying that the latter could allow controlled release of the drug. However, W17 showed a swelling capacity of 318 ± 18% and totally disintegrated after only 90 min, making it unsuitable for vaginal application as the wafer would hydrate quickly to form a free-flowing gel, with poor mucoadhesion and a short residence time on the mucosa.

The W26 and W27 wafers totally disintegrated after 10 min; therefore, their swelling profiles could not be plotted. However, as shown in Figure 3b, the W23 and W25 wafers formed strong gels after hydration and swelling, and only completely disintegrated after 9 h and 12 h, respectively, with a swelling capacity of 101 ± 11% and 439 ± 8%, respectively. W24, on the other hand, lasted until 72 h and exhibited the highest swelling capacity of 697 ± 67%, which makes it a suitable formulation for the controlled release of the drug.

The BLK wafers (W14 and W16) showed a higher % swelling capacity compared to the corresponding DL wafers (W26 and W27) in SVF. This could be attributed to the recrystallization of the drug after freeze-drying in the latter, which affects their swelling capacity [30]. However, other DL wafers (W23, W24, and W25) showed a higher swelling capacity than some (W17, W10, and W13) of the BLK wafers, which could be attributed to the lower CARB concentration and corresponding increasing SA concentration. The statistical analysis showed that there was significant difference (*p* < 0.05) between the swelling capacities of the three DL wafers. This difference was attributed to the changes in the ionic strength of the media, depending on the rate of drug release, which was dependent on the swelling of the wafers and was determined by the initial rate of water uptake (hydration).

The water uptake (swelling) profiles of the wafers were different from the results of a study by Lupo et al. [31], who investigated MTz-loaded vaginal tablets prepared using an ‘entirely S-protected chitosan’ as the mucoadhesive polymer, where a more sustained swelling matching the drug-release profiles was observed. This was related to the stronger mechanical properties of the tablets, which were produced by compression, compared to the wafers prepared by the freezing of polymeric gels, which yielded a more porous micro-structure and a flexible polymer matrix.

#### 3.2.5. Scanning Electron Microscopy (SEM)

**Figure 4:**
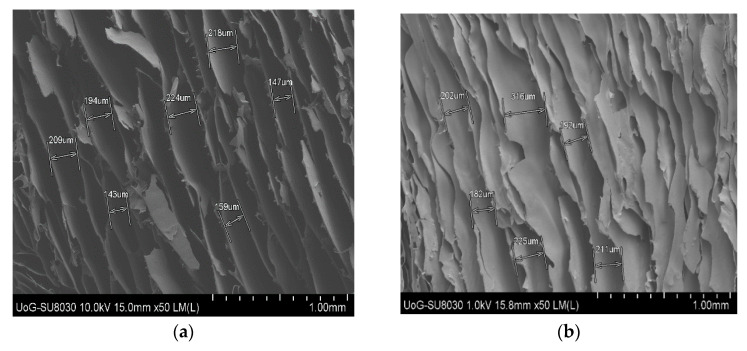

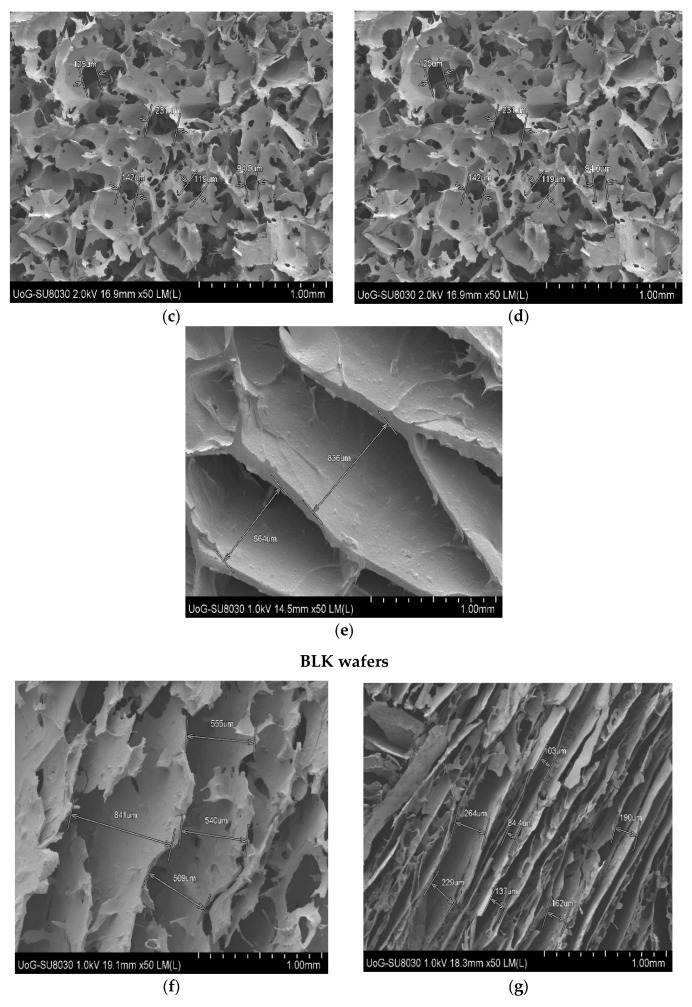

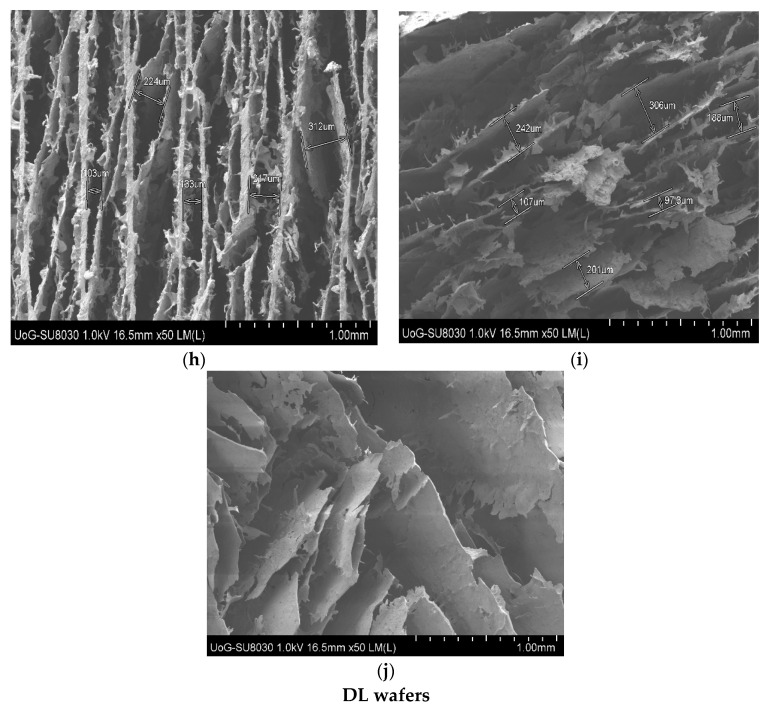
Representative SEM micrographs of optimized BLK and DL wafers. (**a**) W10; (**b**) W13; (**c**) W14; (**d**) W16; (**e**) W17; (**f**) W23; (**g**) W24; (**h**) W25; (**i**) W26; and (**j**) W27.

The SEM microstructure of the wafers and the effect of the HPMC modifier are shown in Figure 4. All the wafers showed a porous microstructure; however, the CARB:CARR wafers showed circular pores, while the CARB:SA wafers showed elongated pores. Different polymer ratios and the presence of HPMC, which has a high affinity for water and affects ice crystal formation during the freeze-drying process, affected the ice crystal size and therefore the pore size, which subsequently affected the rate of hydration, swelling, and mucoadhesion. The DL CARB:SA wafers (W23, W24, and W25) also showed elongated pore structures, as with the corresponding BLK wafers; however, they possessed thicker walls due to the incorporation of the drug as well as the distribution of excess MTz crystals on the walls.

The increase in the thickness of the layers in the CARB:SA wafers confirmed the increase in their swelling capacity. On the contrary, the DL CARB:CARR wafers (W26 and W27) showed flaky leafy structures with thinner walls, which explains the differences observed in the hardness and swelling between the two composite formulations. It has been previously reported that increased porosity can result in a lower hardness because of the reduced interactions between the chains within the polymeric network [32].

#### 3.2.6. Porosity

**Figure 5:**
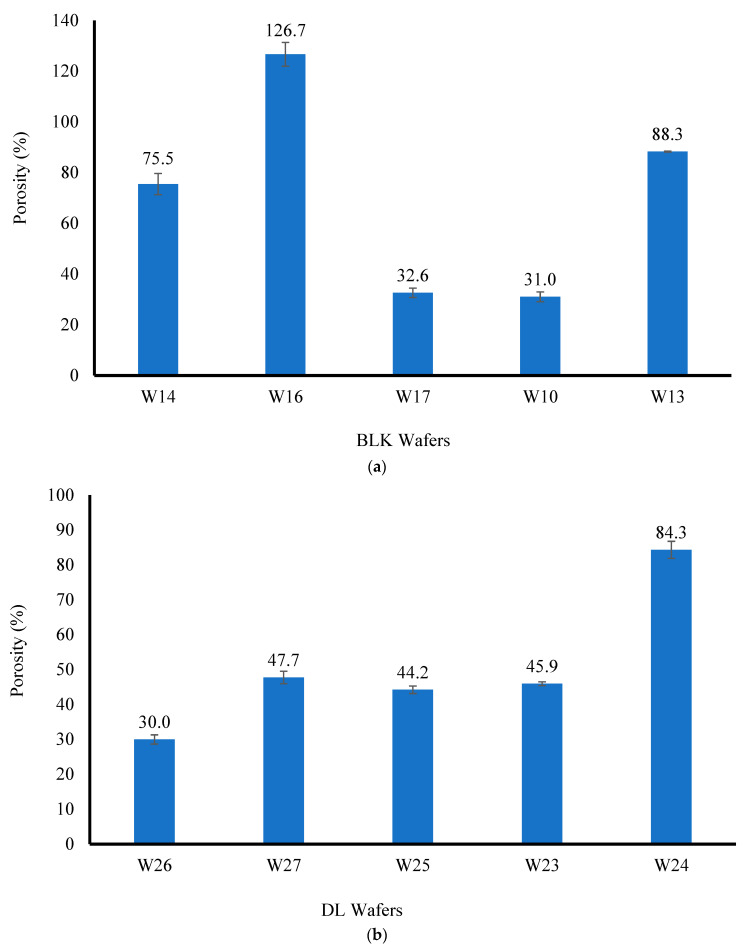
The porosity (%) profiles of BLK (**a**) and DL (**b**) wafers.

The porosity profiles are shown in Figure 5 for the BLK and DL wafers. For the BLK wafers, the presence of HPMC resulted in higher % porosity values, although W13, which contained CARB:SA, also showed high a porosity value of 88.3%.

For the BLK W14 and W16 wafers, the loading of MTz decreased the porosity, while drug loading increased the porosity for the W10, W13, and W17 wafers. It was observed that as the concentration of CARR in the wafers increased (W26 and W27), the % porosity also increased. The highest porosity (%) of the DL wafers was for W24, with a value of 84.3 ± 2.4% due to a higher concentration of SA and a low concentration of CARB, and interestingly, its corresponding BLK wafer had the second highest porosity. The porosity profiles did not directly correlate with the SEM micrographs, which is because SEM shows surface porosity and not internal interconnections. Unlike W24, the porosity results for W25, W26, and W27 appeared to be independent of the composite polymers used, which might be due to the blockage of pore capillaries by MTz, therefore causing the solvent to penetrate less effectively. A statistical analysis of the % porosity for the DL wafers (W23 and W27) showed a significant difference (*p* < 0.05) between them. This may be attributed to the increase in the total solid (polymer and drug) content for W25, which increased in viscosity due to the higher density of the gels.

#### 3.2.7. X-ray Diffraction (XRD)

**Figure 6:**
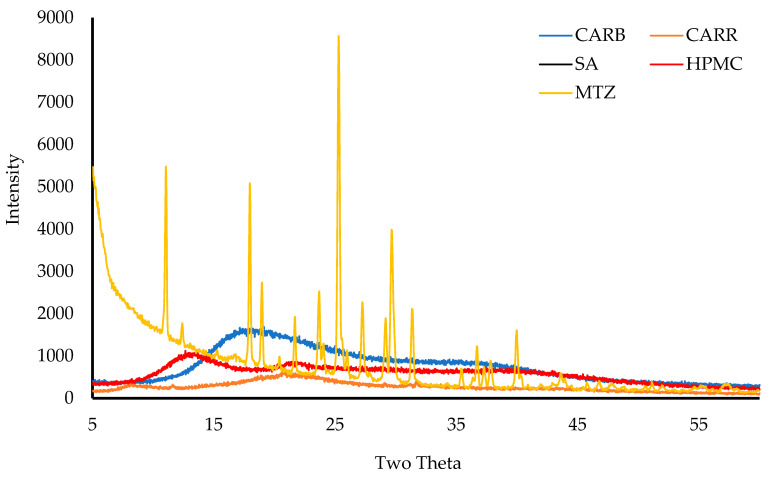
The XRD transmission diffractograms of the pure materials—CARB, CARR, SA, HPMC, and MTz.

Figure 6 shows the transmission diffractograms of pure CARR, SA, CARB, HPMC, and MTz. The CARR, CARB, SA, and HPMC showed amorphous characteristics, however, the CARR showed a small crystalline peak at a 2θ of 23° attributed to inorganic salt impurities from KCl [21].

There were no sharp peaks for CARB, which showed a broad peak from a 2θ of 15° to 25°, confirming its amorphous nature. MTz showed multiple intense sharp peaks between a 2θ of 12° to 35°, confirming that MTz is a solid in crystalline form. The main peaks for MTz observed in this study were compared to the corresponding reference peaks in the International Centre for Diffraction Data (ICDD^®^) database and they matched closely, as shown in Table 6 below.

**Figure 7:**
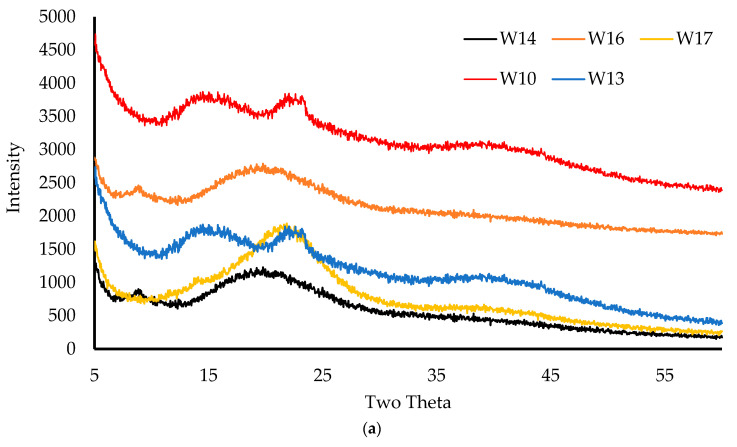

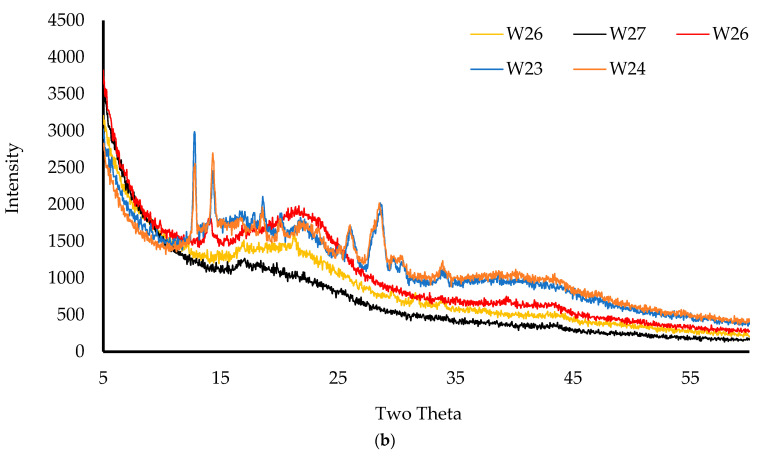
XRD transmission diffractograms of the BLK (**a**) and DL wafers (**b**).

The diffractogram in Figure 7a shows that the BLK wafers were amorphous; however, small peaks at 2θ values of 18° and 40° were observed, and these were the result of the inorganic KCl found in CARR. In addition, there was a broad amorphous peak at 2θ values of 10° and 20°, which was attributed to CARB. Two small crystalline shoulder peaks at 2θ values of 25° and 45° in sequence were observed for 3% CARB:CARR 1:1. These might be due to false peak detection caused by compressing the wafer, which caused the leafy networks to be arranged on top of each other and thus be detected as a false crystalline peak [33].

The diffractogram in Figure 7b confirms the general amorphous nature of the polymeric matrix of the DL wafers, with reduced intensity of the crystalline peaks related to MTz from a 2θ of 12° to 35°. This suggests that most of the drug was molecularly dispersed within the polymeric wafer matrix, with the excess that crystallized on the pore walls (SEM results) detected as crystalline peaks. The W25 wafer had a broad peak at 2θ values of 20° and 25°, and this was due to the presence of PEG. Amorphous formulations provide better hydration and swelling, which also impacts the rate of drug release.

#### 3.2.8. Attenuated Total Reflectance Fourier-Transform Infrared Spectroscopy (ATR-FTIR)

**Figure 8:**
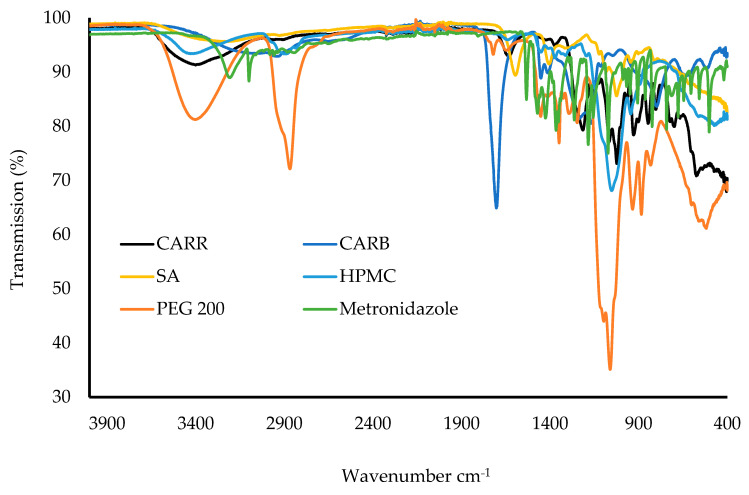
ATR-FTIR spectra of raw materials of CARR, CARB, SA, HPMC, PEG, and MTz.

The ATR-FTIR spectra of the raw materials (CARR, CARB, SA, HPMC, PEG, and MTz) are shown in Figure 8. For CARR, a broad band around 3400 cm^−1^ was attributed to O–H stretching, a sharp peak around 1639 cm^−1^ could be attributed to the glycosidic linkages and the O–C–O bond, and a broad peak at 1260 cm^−1^ showed the presence of the sulfate ester. The peaks at 935 and 1070 cm^−1^ were due to the C–O bonds of 3,6-anhydrogalactose, while the peak at 900 cm^−1^ was due to C–O–SO_4_ present on the C2 of 3,6-anhydrogalactose [34]. For the SA powder, the IR spectrum exhibited characteristic absorption bands for hydroxyl groups (3223 cm^−1^), carboxylate (1405 cm^−1^), and carbonyl (1596 cm^−1^). For the HPMC FTIR spectra, the peak at 3431 cm^−1^ was due to O–H vibrational stretching, while the symmetric stretching mode of hydroxypropyl groups was found at 2895 cm^−1^, in which all the C-H bonds extend and contract in phase. The peak between 1400 and 1350 cm^−1^ suggested the C–O–C of cyclic anhydrides, that at 1300–1250 cm^−1^ was attributed to the C–O–C of cyclic epoxide, and the peak at 1100–1000 cm^−1^ was the result of the stretching vibrations of the C–O–C of the ether functional groups [35]. For CARB, a broad peak was observed at 2936 cm^−1^, which was attributed to the carboxylic acid (COOH) group, with a C=O stretching band at 1703 cm^−1^. For PEG, the absorption bands at 3402 cm^−1^ were due to O–H stretching, that at 2869 cm^−1^ was due to the aliphatic C–H stretching, and the bands at 1453 and 1349 cm^−1^ were due to C-H bending vibrations. MTz showed characteristic peaks at 3457 cm^−1^ (O-H stretching), 3100 cm^−1^ (C–C stretching), 1534 and 1366 cm^−1^ (nitroso N–O stretching), and 1075 and 875 cm^−1^ (C–N stretching).

**Figure 9:**
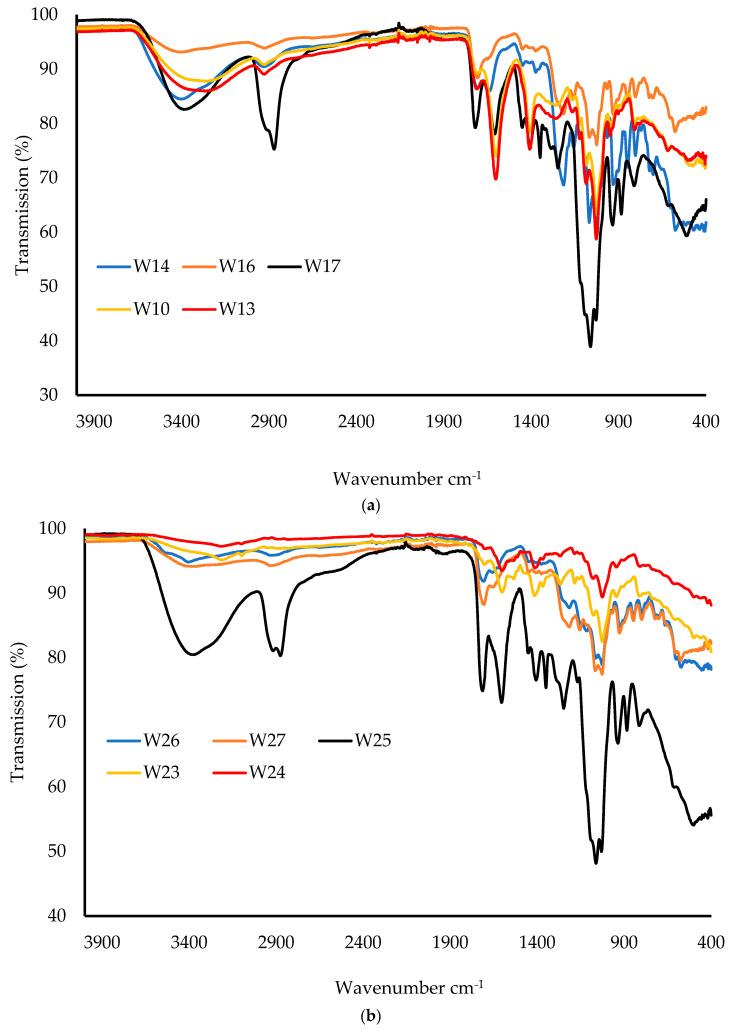
ATR-FTIR spectra of BLK wafers (**a**) and DL wafers (**b**).

Figure 9a shows the ATR-FTIR spectra of the BLK wafers. It was observed that there were no interactions for wafers when compared to the pure materials, as there were no new peaks or significant shifts of the major peaks. For the CARB:CARR wafers, the intensity of the peak at 2936 cm^−1^, corresponding to O–H stretching, increased as the concentration of CARB increased. The peak around 1400 cm^−1^ indicated the C–H bond of the alkane functional group in the CARB:SA-based wafers. The C–C bond in the aromatic ring of SA was expressed as a sharp peak around 1600 cm^−1^. The spectra of the composite CARB:SA wafers containing PEG showed that as the concentration of the total polymer increased, the intensity of the peaks increased, which suggests the formation of strong bonds. The presence of a peak at 3400 cm^−1^ corresponds to the hydroxyl group of the PEG present in the W17–W19 wafers. Figure 9b shows the spectra of the DL wafers, with no shifts in the major peaks found in the starting materials and corresponding BLK wafers. 

#### 3.2.9. In Vitro Drug Dissolution and Release Profiles

Before the drug dissolution studies, the saturation solubility was analyzed for the formulations to determine the volume of dissolution media (SVF) required to maintain sink conditions. Furthermore, based on the physico-chemical tests, especially the hardness and swelling results, only the W23 and W24 wafers were chosen for the in vitro drug dissolution study because the other DL wafers were weak and disintegrated as soon as they made contact with the SVF. It was observed that for the W23 and W24 wafers, the release was 39.6% and 36.9% within 6 h, respectively, gradually increasing over time and remaining constant until almost 100% release was achieved in 72 h for both formulations. This gives a good profile of the controlled release of MTz, and the formulations are expected to maintain antibacterial efficacy over 3 days. However, this is limited by being in vitro, and will therefore need to be verified in an in vivo vaginal infection animal model. Such a model will enable the confirmation of the potential use of these formulations for treating bacterial vaginosis.

**Figure 10:**
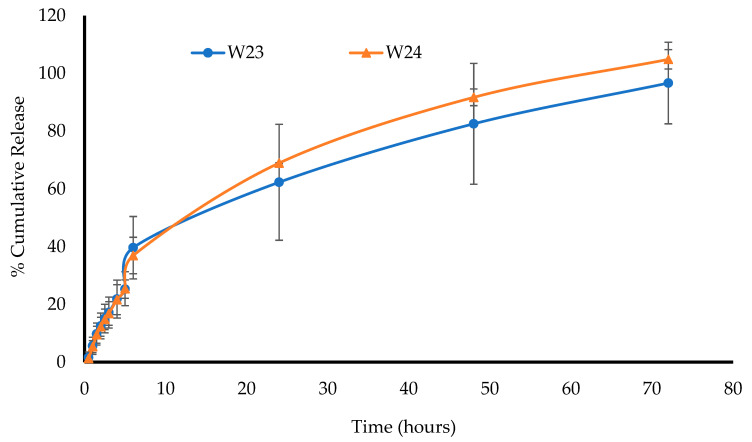
In vitro drug dissolution profiles of MTz (W23 and W24) in SVF at a pH of 4.2 ± 0.1 (mean ± SD, *n* = 3).

Drugs are typically released from a polymer-based formulation via diffusion through the swollen matrix, followed by the erosion of the gel matrix. The release of the drug can be controlled by diffusion, erosion, or a combination of both [36]. When the formulation comes into contact with the dissolution fluid, it hydrates, swells, and finally erodes (dissolution). This was the case for both W23 and W24, as the typical release sequence correlated very well with their plotted release profiles. The dissolution profiles over 72 h for the DL wafers in the SVF at a pH of 4.2 are shown in Figure 10.

In a related study, Badawi et al. [37] reported on MTz-loaded nanoparticles embedded within a Carbopol-based vaginal gel (vaginal emulgel) for the treatment of bacterial vaginosis. The release of the drug from the emulgel was more controlled than the plain gels with no nanoparticles embedded, with the latter achieving 100% cumulative release within 2 h while the emulgel released 82% in 24 h. Interestingly, the two optimized wafers in this study (W23 and W24) demonstrated 62 and 68% release over 24 h, respectively, and achieved almost 100% release over 72 h. This was attributed to the wafers maintaining their structural integrity after swelling, which then controlled the drug release over a longer period. This implies their potential for a more prolonged application and therefore a lower dosing frequency, which would be more convenient. Jalil et al. [36] prepared mucoadhesive vaginal films using ‘S-protected gellan’ loaded with MTz and evaluated their in vitro drug release and antimicrobial action against *E. coli* as a model bacterial organism. Their results showed relatively rapid release of MTz from the films, achieving between 70 and 95% cumulative release within 3 h compared to the results from this study, which showed a far better controlled release. The difference can be attributed to the physical architecture, with the films being thin and therefore allowing easier access of the SVF compared to the rocket-shaped wafers, which took longer for complete swelling and thus controlled the drug diffusion. Interestingly, in a related study by Lupo et al. [31] who formulated vaginal tablets from ‘S-protected chitosan’, the formulations released 100% of MTz within 6 h, which was twice as controlled as the ‘S-protected gellan gum’, but still less controlled than the freeze-dried wafers prepared in this study. This confirms our hypothesis that freeze-dried wafers present a potential platform for prolonged retention and controlled drug release for treating vaginal infections.

##### Kinetic Mechanism of Drug Release

**Figure 11:**
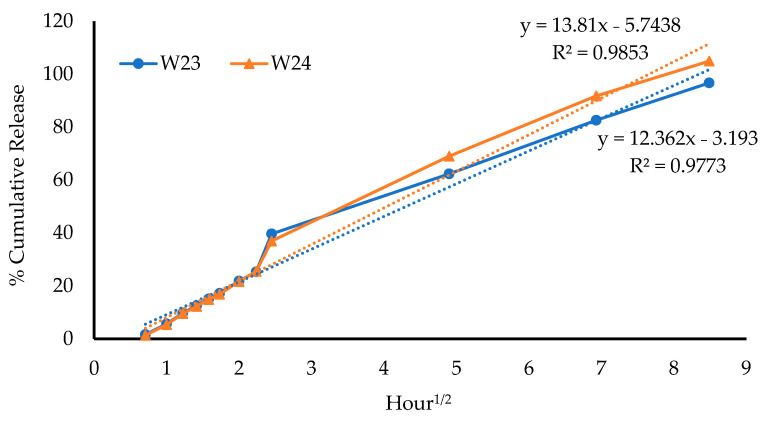
Kinetic curves calculated using the Higuchi equation, showing high correlation coefficients for both formulations.

The release parameters of the DL wafers are summarized in Table 7, and based on the R^2^ values, the MTz release data best fit the Higuchi equation for both W23 and W24, as evidenced by the calculated kinetic curves shown in Figure 11 below. This suggests that the release of MTz from wafers was due to diffusion from the swollen wafer matrix. The ‘*n*’ value in the Korsmeyer–Peppas equation describes the release exponent, which is employed to elucidate the drug-release mechanism from swellable matrix systems. The calculated *n* values from the Korsmeyer–Peppas equation for both W23 and W24 were 0.72 and 0.77, respectively (Table 7), which was attributed to anomalous (non-Fickian) diffusion as defined by *n* values between 0.45 and 0.89 (0.45 < *n* < 0.89).

### 3.3. In Vitro Antimicrobial Testing

#### 3.3.1. Minimum Inhibitory Concentration (MIC) and Minimum Bactericidal Concentration (MBC)

The MIC of an antimicrobial agent is the lowest concentration that inhibits the growth of a particular microorganism after incubating for 24 h. On the other hand, the MBC is the lowest concentration of an antimicrobial agent that can inhibit microbial growth by 99.9% in CFU/mL relative to the control sample [38,39]. The MIC value of MTz was determined to be 4 mg/mL for *E. coli* and the MBC range (0.1–10 mg/mL) of MTz was evaluated further to establish the exact MBC value. The results showed that MTz killed 99.9% of *E. coli* cells (1 × 10^6^ CFU/mL) at a concentration of 4 mg/mL compared to the control (drug-free culture) (2 × 10^8^ CFU/mL).

#### 3.3.2. Antibacterial Activity of MTz-Loaded Wafers

**Figure 12:**
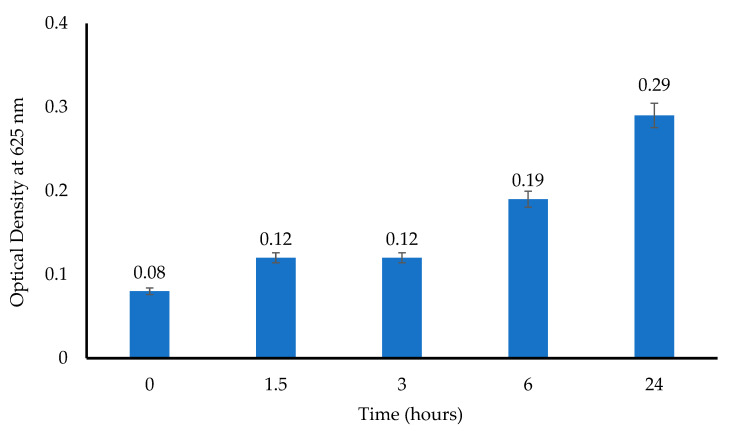
Bacterial growth (optical density) profiles over 24 h (*n* = 3 ± SD).

The principle behind the turbidimetric assay is based on the interaction between the drug in the wafers and the test microorganism (*E. coli*), with a subsequent change in the turbidity of the bacterial suspensions. Figure 12 shows that the absorbance values of *E. coli* gradually increased between 3 and 24 h, which confirmed the growth of microorganisms.

**Figure 13:**
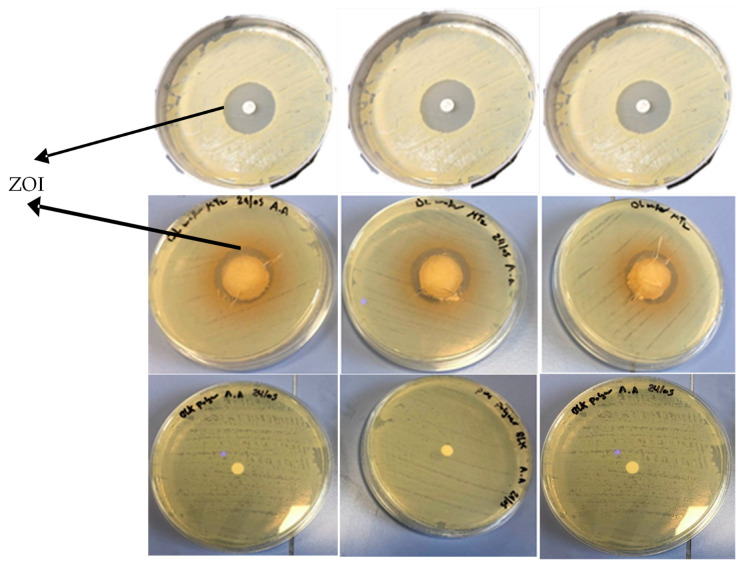
Zones of inhibition (ZOI) of pure MTz as positive control (**top**), DL wafers (**middle**), and BLK wafers (**bottom**).

**Figure 14:**
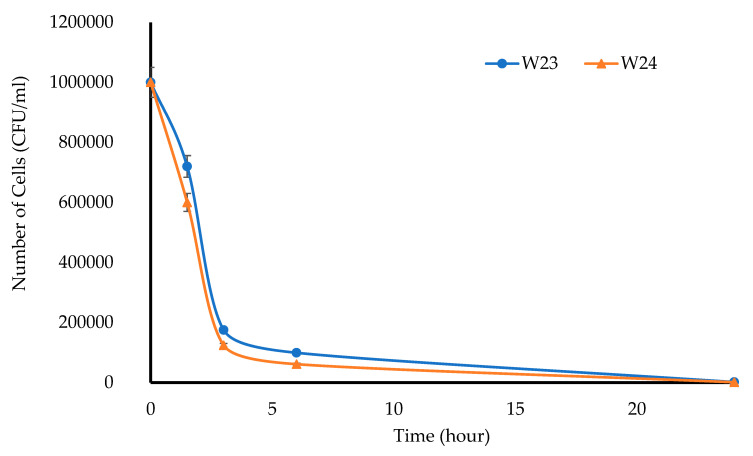
Rate of bacterial inhibition after treatment with DL wafers against *E. coli*. (*n* = 3 ± SD).

The ZOI assay attempts to simulate the use of the antimicrobial product clinically, and in this case, predict the wafers’ ability to eliminate bacteria completely or retard their ability to grow. An infected vaginal mucosal surface was simulated using the inoculated BHI agar plates. To be able to exert its antibacterial action, the DL wafers would have to absorb moisture from the agar plates colonized with the *E. coli*, swell, and subsequently trigger the release of MTz from the swollen matrix onto the agar to exert its antimicrobial effect. Figure 13 shows the inhibitory zones of the BLK and DL wafers placed on the *E. coli* cultures. The BLK wafers did not show any inhibition zones, while the MTz-loaded wafers showed clear ZOIs (black arrows), and this was confirmed by the turbidimetric and time-kill assays (Figure 14). The pure MTz soaked onto filter papers showed the strongest inhibitory effect (ZOI, 28.77 ± 0.12 mm) against the *E. coli*, which is not surprising since no swelling step was involved compared to the MTz-loaded wafers. The results confirmed the ability of the wafers to release the loaded MTz to exert both inhibitory and bactericidal action against the model antimicrobial agent and showed their potential for use in treating vaginal infections caused by bacteria.

This was similar to the results of the studies conducted by Lupo et al. [31] and Jalil et al. [36], both of which showed strong zones of inhibition for the MTz released from vaginal films and tablets prepared using S-protected natural polymers against *E. coli*. This notwithstanding, it will be essential to validate the in vitro result using an in vivo animal model to confirm the potential application of these formulations for treating vaginal bacterial infections in a clinical setting.

## 4. Conclusions

This project aimed to develop, characterize, and optimize polymer-based freeze-dried wafers for the delivery of antimicrobial agents (using MTz as a model antibacterial drug) via the vaginal mucosa over a prolonged period to treat local infections. Composite wafers were initially formulated using GRAS mucoadhesive polymers, which were chosen depending on their ability to attach to the vaginal mucosa and achieve controlled release of the drug. Composite wafers comprising two natural polymers, CARR and SA, did not yield the expected optimal functional physical properties, especially hardness. Therefore, a synthetic polymer, CARB, was added to the formulations to improve the physical properties. The combination of CARB with CARR and CARB with SA at optimal concentrations showed an improved cone (rocket) shaped structure for easy vaginal insertion, improved porosity, and improved mechanical properties, which were then selected for loading the drug. The XRPD results indicated the amorphous nature of the BLK wafers; however, MTz maintained its crystalline nature in the DL wafers. The SEM micrographs showed a highly porous microstructure for the BLK wafers, while the MTz-loaded wafers showed a more compact structure with dispersed drug crystals on the pore walls. Based on the hardness profiles, W23 and W24 were chosen for the in vitro drug dissolution study. The drug release was controlled over 72 h, suggesting the potential to achieve the prolonged release of the drug, which would reduce the need for regular insertion, thereby reducing the incidence of non-compliance. The MTz-loaded optimized wafers also showed better inhibition of the model infection-causing bacteria (*E*. *coli*) compared to the BLK wafers. Overall, the MTz CARB:SA wafers show potential for vaginal mucosal delivery to treat local bacterial infections within the vaginal cavity. Though interesting, the results from the functional characterization are limited by being in vitro. Further studies, including testing mucoadhesion and drug permeation using an ex vivo (e.g., porcine) vaginal mucosal model, as well as an in vivo bioadhesion and antibacterial infection model, will be required in the future.

## Figures and Tables

**Table 1:** Blank (BLK) composite CARB:CARR and CARB:SA gels showing the different ratios of the polymers and total polymer concentrations.

Wafer #	Combined Polymers	Polymer Ratios	Total Polymer Concentration (% *w/v*)
W1	CARB:CARR	1:3	3
W2	CARB:CARR	3:1	3
W3	CARB:CARR	1:1	3
W4	CARB:SA	1:4	3
W5	CARB:SA	4:1	3
W6	CARB:SA	1:1	3
W7	CARB:SA	1:4	4
W8	CARB:SA	4:1	4
W9	CARB:SA	1:1	4
W10	CARB:SA	1:4	8
W11	CARB:SA	4:1	8
W12	CARB:SA	1:1	8
W13	CARB:SA	1:9	8

**Table 2:** Blank (BLK) composite CARB:CARR gels (6% *w/v*) modified with HPMC showing different ratios of the polymers.

Wafer #	Combined Polymers	CARB:CARR Concentration (% *w/v*)	CARB:CARR Ratio	HPMC Concentration (% *w/v*)	Total Polymer Concentration (% *w/v*)
W14	CARB:CARR	6	1:3	1	7
W15	CARB:CARR	6	3:1	1	7
W16	CARB:CARR	6	1:1	1	7

**Table 3:** Blank (BLK) composite CARB:SA gels (8% *w/v*) loaded with 8% PEG with or without HPMC showing different ratios of the polymers.

Wafer #	Combined Polymers	Concentration of CARB:SA (% *w/v*)	CARB:SA Ratio	PEG Concentration (% *w/v*)	HPMC Concentration (% *w/v*)	Total Polymer Concentration (% *w/v*)
W17	CARB:SA	8	1:2	8	0	16
W18	CARB:SA	8	2:1	8	0	16
W19	CARB:SA	8	1:1	8	0	16
W20	CARB:SA	8	1:2	8	1	17
W21	CARB:SA	8	2:1	8	1	17
W22	CARB:SA	8	1:1	8	1	17

**Table 4:** Drug loaded (DL) composite CARB:SA and CARB:CARR gels at different concentrations and ratios with or without PEG/HPMC.

Wafer #	Combined Polymers	CARB:SA or CARB:CARR (% *w/v*)	CARB:SA/CARB:CARR Ratio	PEG/HPMC Content (% *w/v*)	Total Polymer Concentration (% *w/v*)	MTz Concentration (% *w/v*)
W23	CARB:SA	8	1:4	0	8	0.75
W24	CARB:SA	8	1:9	0	8	0.75
W25	CARB:SA	8	1:2	8 (PEG)	16	0.75
W26	CARB:CARR	6	1:3	1 (HPMC)	7	0.75
W27	CARB:CARR	6	1:1	1 (HPMC)	7	0.75

**Table 5:** Various equations describing relationship between drug release and time.

Plot Parameters	Kinetic Mechanism/Model
Cumulative % drug release against time	Zero-order
Log cumulative of % drug remaining against time	First-order
Cumulative % drug release against square root of time	Higuchi
Log cumulative % drug release against log of time	Korsmeyer–Peppas

**Table 6:** The main reference peaks from the International Centre for Diffraction Data (ICDD^®^) for MTz (ICDD PDF 38-1556) compared to those obtained from the current study in 2θ.

ICDD Peak Position	Peak Position for Current Study
12.28°	12.29°
13.89°	13.89°
16.25°	16.25°
17.31°	17.33°
18.13°	18.13°
19.58°	19.57°
21.61°	21.62°
24.77°	24.78°
25.43°	25.42°
27.48°	27.50°
28.11°	28.10°
29.41°	29.42°
30.02°	30.02°
33.46°	33.47°

**Table 7:** Release parameters of DL wafers in SVF developed by fitting experimental drug dissolution (release) data to different kinetic equations.

Formulations	Zero-Order		First-Order		Higuchi		Korsmeyer–Peppas	
	R^2^	K_o_ (hour)	R^2^	K_1_ (hour ^1^)	R^2^	K_H_ (hour ^1/2^)	R^2^	*n*
W23	0.8967	1.2998	0.5212	0.0171	0.9773	12.3620	0.9027	0.72
W24	0.9081	1.4541	0.5212	0.0171	0.9853	13.8100	0.9034	0.77

## Data Availability

Not applicable here.
